# Items

**Published:** 1904-08

**Authors:** 


### ITEMS.

---

The Philippine government exposition at the Saint Louis World's Fair is the largest single exhibit on the grounds. It occupies 47 acres in which are housed 75,000 catalogued exhibits, as well as 1,100 representatives of the different peoples of the islands. The sum of $1,000,000 has been appropriated for the purpose of collecting and installing this exhibit, four-fifths of which is borne by the Philippine insular government.

It comprises, in general, the walled city and its approach, the bridge of Spain; the dwellers on Arrowhead Lake; the agricultural exhibit; the Plaza Santa Cruz; a demonstration of educational progress, including a Philippine school in active operation; a replica of the capitol building of the Philippines; an exhibit of the tropical hard woods; an ethnology exhibit; a fisheries exhibit; an exhibit of mineral wealth, and groups of men and women selected from various tribes, including Negritos, Igorrotes, Moros, and others, all with their native surroundings. Besides these, many other items of interest that are too numerous and varied to justify mention in this brief article, are offered for the instruction and entertainment of visitors. All Americans interested in the progress making by our country in developing and improving its insular possessions should visit this marvelous collection of people and material.

Two commodious restaurants offer food and drink to the visiting public at reasonable rates; seats are scattered throughout the grounds, the use of the toilet rooms is free, cool, filtered drinking water is offered gratuitously, the Constabulary Band of eighty pieces gives a free concert twice daily, and no pains or expense have been spared to make the stay of the visitor to the Philippine Exposition interesting, pleasant and profitable.

---

The Doctors' Chronological Lactopeptine Calendar is the name given to a new almanac issued by the New York Pharmacal Association, Yonkers.

---

Battle & Company, of Saint Louis, have issued pamphlets one and two, of the series of twelve illustrations of intestinal parasites, these relating to the tenia saginata.

---

The World's Fair Catalogue.—The official catalogue of the exhibits of the Saint Louis Exposition reveals the tremendous scope and wonderful industrial development of the world as illustrated therein. In the first edition, which is sent by mail postpaid to any address on receipt of $5.33 by the publishers, more than 60,000 exhibitors are listed. In the full catalogue, which will contain in addition the exhibits of the Philippines and live stock, the exhibitors will reach 150,000. The first edition can be had by addressing The Official Catalogue, World's Fair, Saint Louis.

